# Medical Association of Georgia

**Published:** 1895-03

**Authors:** 


### Editorial.

MEDICAL ASSOCIATION OF GEORGIA.

The forty-sixth session of the Medical Association of Georgia will be held in Savannah on April 17, 18 and 19. We are always glad when the time comes for the association to meet; it brings together the best element of the profession of the State, gives them the opportunity to interchange ideas, both through the reading of the papers and through personal conversations, and to many of those present affords the only vacation they take from a most arduous profession. The physicians are the only class we know who work three-hundred and sixty-five days in a year and yet hold themselves ever ready to work night after night if need be. Certainly such men honestly earn a few days rest, and we think this feature of the association is not one of its least benefits. The doctors who attend, go home not only with new ideas, but with rested brains and bodies, ready for long rides and sleepless nights. We city physicians do not fully appreciate the hardships of our country brethren; we do less work and are better paid. Let us then insist that they come to Savannah next month, that they come prepared to have a good time as well as to give us the benefit of their experiences rich in valuable lessons gained under the most adverse conditions.

No more charming place could have been selected for the meeting than old Savannah, famed for her hospitality.

The preliminary programme contains a large number of papers on many subjects of interest and all the indications point, in spite of hard times, to a full attendance and an interesting programme.

---

The forty-sixth annual session of the Medical Association of Georgia will take place in Savannah, Ga., April 17, 18 and 19, 1895.

The following is a portion of the preliminary programme:

Some Observations with Regard to the Treatment of Typhoid Fever.—A. C. Davidson, M. D., Sharon.

The Cause and Treatment of Corneal Ulcers.—S. Latimer Phillips, M. D., Savannah.

Traumatic Wounds of the Eye and their Treatment.—T. E. Mitchel, M. D., Columbus.

The Topical Treatment of Chronic Circumscribed Eczema.—Bernard Wolff, M. D., Atlanta.

Some Grave Cases in Obstetric Practice, with Especial Reference to the Treatment.—C. H. Richardson, M. D., Montezuma.

Management of Obstetric Cases, Report of Cases.—Thos. D. Love, M. D., Atlanta.

Nasal Sprays: What Solution to Use.—George Brown, M., D., Atlanta.

Galvanism in Office Practice.—J. C. LeHardy, M. D., Savannah.

Obstetrics in Vienna, Austria.—Wm. B. Gilmer, M. D Macon.

Uranalysis.—Louis H. Jones, M. D., Atlanta.

A Duty of the Association.—F. M. Ridley, M. D., LaGrange.

The Surprises We Meet in Abdominal Surgery.—Seth N. Jordan, M. D., Columbus.

Something New in the Treatment of Women after Confinement.—R. J. Nunn, M. D., Savannah.

The Proper Care of School Children's Eyes; The Necessity.—Dunbar Roy, M. D., Atlanta.

Backbone as a Mental Antiseptic.—J. S. Wimberly, M. D., Sanford.

Gonorrhœa, its Effects and Treatment in the Female.—J. W. Bailey, M. D., Gainesville.

The Apostoli Treatment of Uterine Fibroids.—E. R. Corson, M. D., Savannah.

The Uses of Strychnine in Obstetric Practice.—Jos. Eve Allen, M. D., Augusta.

Nephroptosis, its Causation, Treatment, and its Relation to many Obscure Symptoms in Women (continued).—W. W. Stewart, M. D., Columbus.

One Hundred Consecutive Cases of Labor.—H. McHatton, M. D., Macon.

A Remarkable Case of Ruptured Ectopic Gestation; Late Operation; Recovery.—Geo. H. Noble, M. D., Atlanta.

Placenta Praevia, Report of Case.—M. L. Currie, M. D., Ailey.

A Rare Intestinal Tumor.—L. G. Hardman, M. D., Harmony Grove.

Reports of Several Interesting Operations.—W. S. Armstrong, M. D., Atlanta.

Graves Disease, with Cases.—J. M. Hull, M. D., Augusta.

Ligation of External Carotid Artery as Preliminary to and Coincident with Operations upon the Jaws, with Report of Case.—Wm. Perrin Nicolson, M. D., Atlanta.

The Limits of Conservatism in Pelvic Pus-Cases.—Ross P. Cox, M. D., Rome.

Hip and Shoulder-joint Amputation, with Report of Cases. —F. M. Ridley, M. D., LaGrange.

A Postscript to My Paper on Scopolomine as a Mydriatic.—A. G. Hobbs, M. D., Atlanta.

Mind and Body.—Chas. S. Webb, M. D., Atlanta.

A Ready Method of Resuscitation in Suspended Animation —P. L. Hilsman, M. D., Albany.

A Case of Cancer of Pancreas, with Autopsy.—Valentine H. Taliaferro, M. D., Atlanta.

Mental Suggestion as an Aid in the Treatment of Morphinomania; Cases in Evidence.—Samuel H. Green, M. D., Oakdale.

Wounds from Explosives: Their Treatment.—T. D. McKown, M. D., Chickamauga.

Obstetrics as Practiced in the Mountains of North Georgia. —T. M. Greenwood, M. D., Mineral Bluff.

Title to be Given.—C. D. Hurt, M. D., Atlanta.

A Syllabus of the Medical and Surgical History of Georgia. —L. B. Grandy, M. D., Atlanta.

Sympathetic Affections of the Eye, with Report of Cases.—Jas. H. Shorter, M. D., Macon.

Flap-Splitting Methods in Perineal Work.—R. R. Kime, M. D., Atlanta.

Appendicitis: a Brief Review of my Individual Experience.—Floyd W. McRae, Atlanta.

Laparotomy During Pregnancy.—Virgil O. Hardon, Atlanta.

A Report of My Surgical Work for the Six Months Ending April 15th, with Some Deductions Therefrom.—J. B. S. Holmes, M. D., Atlanta.

